# CRISPR/Cas-Dependent and Nuclease-Free *In Vivo* Therapeutic Gene Editing

**DOI:** 10.1089/hum.2021.013

**Published:** 2021-03-17

**Authors:** Ishani Dasgupta, Terence R. Flotte, Allison M. Keeler

**Affiliations:** Department of Pediatrics, Horae Gene Therapy Center, University of Massachusetts, Worcester, Massachusetts, USA.

**Keywords:** CRISPR/Cas9, *in vivo*, gene editing, gene therapy, HDR pathway, AAV vectors, monogenic

## Abstract

Precise gene manipulation by gene editing approaches facilitates the potential to cure several debilitating genetic disorders. Gene modification stimulated by engineered nucleases induces a double-stranded break (DSB) in the target genomic locus, thereby activating DNA repair mechanisms. DSBs triggered by nucleases are repaired either by the nonhomologous end-joining or the homology-directed repair pathway, enabling efficient gene editing. While there are several ongoing *ex vivo* genome editing clinical trials, current research underscores the therapeutic potential of CRISPR/Cas-based (clustered regularly interspaced short palindrome repeats-associated Cas nuclease) *in vivo* gene editing. In this review, we provide an overview of the CRISPR/Cas-mediated *in vivo* genome therapy applications and explore their prospective clinical translatability to treat human monogenic disorders. In addition, we discuss the various challenges associated with *in vivo* genome editing technologies and strategies used to circumvent them. Despite the robust and precise nuclease-mediated gene editing, a promoterless, nuclease-independent gene targeting strategy has been utilized to evade the drawbacks of the nuclease-dependent system, such as off-target effects, immunogenicity, and cytotoxicity. Thus, the rapidly evolving paradigm of gene editing technologies will continue to foster the progress of gene therapy applications.

## Introduction

Gene editing has emerged as one of the most revolutionary breakthroughs in the field of biomedical sciences over the past decade. The technological advancements developed by scientists have enabled precise and targeted manipulation of the genome. Gene editing approaches entail a site-specific modification of a gene by its deletion, replacement, or correction, thus producing the desired therapeutic effect.

Fundamentally, site-specific modification of genetic information at the DNA level requires two essential components: first, a sequence-specific DNA recognition and binding domain, and second, an effector domain that initiates DNA cleavage near the binding site. A double-stranded break (DSB) by a sequence-specific endonuclease activates the cell's endogenous DNA repair mechanisms, subsequently modifying the desired sequence.^1–3^ Two major repair pathways used by the cell to repair the nuclease-induced DSB are nonhomologous end-joining (NHEJ) and homology-directed repair (HDR).^4–6^ Depending on the DSB repair pathway, the outcome may be inactivation of the targeted locus by insertions or deletions (“indels”) introduced by NHEJ^7^ or insertion of a new sequence by HDR from an exogenous DNA template. In the HDR pathway, the donor DNA has homology arms with sequences identical to the region surrounding the DSB, enabling precise correction or replacement of the original gene.^4^ These alternations are triggered by engineered nucleases that induce a double-stranded break (DSB) in the desired genomic locus, leading to activation of efficient DNA repair mechanisms present in all organisms. Here we mention the different nuclease-mediated platforms, focusing on the CRISPR/Cas (clustered regularly interspaced short palindrome repeats-associated Cas nuclease) system for gene editing.

CRISPR has the potential to be used directly in patients, *in vivo* or *ex vivo*, for therapeutic gene editing. *In vivo* gene editing involves gene modification *in situ* by the direct delivery of CRISPR/Cas9 to target cells. Some parameters need to be considered for recognizing the efficacy and safety of therapeutic *in vivo* gene editing, discussed in the later sections of this article. Ideally, the carriers for delivering CRISPR/Cas directly to target cells should be nonimmunogenic, with minimal cytotoxicity. Only target cells harboring a mutated gene should be edited using CRISPR/Cas components; nonspecific targeting of the normal cells may adversely affect their physiological function. Off-target effects associated with CRISPR/Cas need to be restricted to prevent insertional tumorigenesis. Finally, a high editing efficiency may be required to obtain clinically relevant levels for therapeutic gene editing. Therefore, selection of an optimal CRISPR/Cas system and the appropriate delivery vector is imperative for precise and robust gene editing *in vivo*. In this review, we focus on the nuclease-dependent (CRISPR/Cas) HDR-based editing *in vivo* to treat human monogenic diseases, briefly evaluate the hurdles and mitigation strategies coupled with *in vivo* delivery, and discuss nuclease-free editing as an alternate gene targeting approach.

## Nuclease-Dependent Platforms for Gene Editing

**Figure 1.**
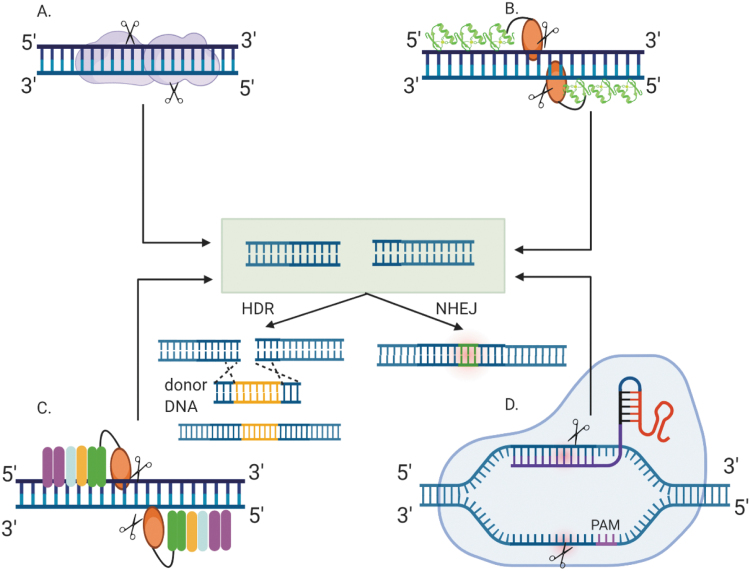
Different programmable nucleases for targeted gene editing. **(A)** Meganucleases belonging to the endonuclease family form a homodimer to exhibit nuclease activity and have a very long target recognition sequence. **(B)** ZFN modules (*green*) linked in tandem are fused to the *Fok*I nuclease (*orange*) with a conserved linker (*black*). **(C)** TALENs are designed by fusing their TALE modules to *Fok*I nuclease, similar to ZFNs. Each TALE module recognizes only one nucleotide base, whereas each ZFN module recognizes three bases. Two distinct ZFNs and TALENs bind to specific sites at opposite DNA strands (*blue*) and *Fok*I dimer cleaves the DNA at the targeted locus. **(D)** CRISPR/Cas system contains an sgRNA and a Cas protein (*light blue*). An ∼20 nucleotide spacer region (*purple*) of the sgRNA recognizes the target DNA to be modified. A PAM sequence (*pink*) acts as a binding signal for the Cas protein. Once the spacer region of the gRNA recognizes and binds to the target DNA, the Cas endonuclease undergoes a conformational change to induce a DSB at the target site. The DSB can be repaired by the host's DNA repair pathways, either by the non-NHEJ or HDR. The NHEJ pathway results in small indels (*green*) in the target DNA and in most cases causes disruption of the mutated/defective gene. HDR pathway requires the presence of an exogenous donor template encoding the desired edit in addition to the sequence homologous to the regions flanking the genomic target and results in high-fidelity and precise gene editing. CRISPR/Cas, clustered regularly interspaced short palindrome repeats-associated Cas nuclease; DSB, double-stranded break; gRNA, guide RNA; HDR, homology-directed repair; indels, insertions/deletions; NHEJ, nonhomologous end-joining; PAM, protospacer-adjacent motif; sgRNA, single-guide RNA; TALENs, transcription activator-like effector nucleases; ZFN, zinc-finger nuclease. Created with BioRender.com

Nuclease-based platforms include meganucleases, zinc-finger nucleases (ZFNs), transcription activator-like effector nucleases (TALENs), and the more recent CRISPR/Cas9 that can be engineered to target the genomic locus of interest (Fig. 1). Meganucleases or homing endonucleases recognize long DNA sequences to trigger a DSB. They were among the first to be reengineered for novel target site recognition using structure-based design and protein engineering approaches.^1,8^ However, the process of designing meganucleases for therapeutic gene editing is laborious, thereby limiting its use. ZFNs consist of a zinc finger DNA binding domain that determines specificity and a nuclease domain derived from a restriction endonuclease, *Fok*I.^9^ A pair of ZFNs are designed for each target site for the *Fok*I domains to dimerize, rendering the nuclease domain catalytically active. Using a wide array of approaches such as phage-based selection, bacterial-based selection, and modular assembly, ZFNs have been constructed to target a diverse range of sequences for gene editing.^10–14^ Engineered ZFNs have exhibited promise in enhancing targeted homologous recombination (HR) in human cells,^15^ as well as therapeutic gene editing for cystic fibrosis,^16^ sickle cell anemia,^17,18^ and human immunodeficiency virus (HIV).^19–21^ SB-FIX by Sangamo Therapeutics is an *in vivo* gene therapy treatment that uses ZFN-based editing to deliver the correct copy of factor IX (FIX) gene for treating hemophilia B. Another ZFN-based *in vivo* gene editing therapy developed by Sangamo Therapeutics, SB-913, entered the first clinical trial for treating Hunter's syndrome.^22^ A similar *Fok*I nuclease-based editing platform, TALENs, derived from TAL effector proteins also demonstrated therapeutic gene editing potential.^23,24^ This technology has been effectively used to mitigate the HIV coreceptor, *CCR5* gene,^23^ and manipulate immune cells for cancer treatment.^25^ Despite the targeted gene editing efficacy of ZFNs and TALENs, the difficulty in cloning and reengineering them for each target site has limited their widespread use. The advent of the CRISPR/CRISPR-associated protein (Cas) technology, which is far more robust and flexible compared with the existing nucleases, paved the way for new possibilities in therapeutic gene editing.

## CRISPR/Cas Technology

This powerful, multiplexed tool first studied as part of the bacterial adaptive immune system consists of a protein (Cas) and an RNA (crRNA and tracrRNA) component. A CRISPR/Cas locus is made up of *Cas* genes and a CRISPR array consisting of repetitive sequences interspaced by variable DNA bases, called spacers. These spacers serve as a “snapshot” of the invader's mobile genetic elements acquired during a previous infection. During future infections, this stored “snapshot” mediates recognition and protection against foreign cognate viruses or plasmids.^26,27^ The CRISPR/Cas-mediated immune response, based on sequence-specific targeting of foreign nucleic acids, is divided into three main stages. The first stage of immune response elicited by the CRISPR system is termed as the acquisition stage, in which DNA fragments from invading viruses are introduced into the CRISPR locus of the host as spacers. The second stage, known as the expression stage, marks transcription of the CRISPR array containing spacers into pre-CRISPR RNA (pre-crRNA), followed by processing it by Cas proteins to mature crRNAs. A noncoding *trans*-activating CRISPR RNA (tracrRNA) is essential for crRNA processing and binding to Cas protein in type II CRISPR systems. The mature crRNA acts as a guide that can recognize invading foreign DNA and direct the Cas protein, thereby mediating target cleavage. During the final interference stage, the crRNA enables target recognition and Cas proteins cleave the foreign DNA, conferring protection to the host cell.^28–30^

Seminal work in the field has shown that this CRISPR/Cas system can be programmed to cleave host DNA in a diverse range of species, thereby enabling gene editing for a multitude of biomedical applications. A variety of CRISPR/Cas systems have rapidly evolved, resulting in their structural and functional diversity. CRISPR/Cas systems are divided into two classes that are each subdivided into three types and various subtypes. The class 1 CRISPR systems (type I, II and IV) present in bacteria and archaea are thought to be evolutionary ancestrally. Their effector complexes consist of multiple Cas protein subunits. Contrary to this, the class 2 CRISPR systems (type II, V, VI) are mostly restricted to bacteria and their effector complex is made up of a single multidomain Cas protein.^31,32^ The type II CRISPR/Cas system has emerged as the most widely used and robust nuclease for genome editing studies.^32^ This RNA-guided type II complex consists of a Cas9 endonuclease and a guide RNA (gRNA). The gRNA constitutes an ∼20-nucleotide crRNA complementary to the target DNA and a scaffold sequence required for Cas binding namely, tracrRNA. A breakthrough discovery showed that it is feasible to fuse the crRNA and tracrRNA into a single chimeric gRNA, which confers specificity to the CRISPR/Cas system for targeted gene editing. In addition, a protospacer-adjacent motif (PAM) sequence immediately downstream of the target site also determines the specificity of this system and serves as a binding signal for the Cas protein. Cas nucleases isolated from different bacteria recognize respective PAM sequences. The most commonly used and well-characterized Cas9 endonuclease is from the bacterium, *Streptococcus pyogenes*, which requires a 5′-NGG-3′ PAM sequence immediately downstream of the target site for binding. Cas9 and gRNA form a ribonucleoprotein (RNP) complex, facilitated by the gRNA scaffold (tracrRNA), while the spacer region (crRNA) is free to interact with the target DNA. Once the RNP complex binds to the putative target DNA, the gRNA anneals to the target and Cas9 undergoes a conformational change; its HNH nuclease domain cleaves the target strand at approximately three to four nucleotides upstream of the PAM sequence and the RuvC-like nuclease domain cleaves the nontarget strand resulting in a DSB at the desired genomic locus.^28,33–35^ The DSBs are repaired either by NHEJ or HDR pathways, as mentioned earlier. Thus, by altering the synthetic gRNA sequence to bind any desired target, the Cas9 protein can be utilized as a robust platform for precise genome targeting. Wild-type Cas9 nuclease variants generated by mutating either of the two nuclease domains function as a nickase Cas9 (nCas9) that cleaves a single strand of DNA. This feature helps in enhancing Cas9-based gene editing specificity.^36,37^ When both the HNH- and RuvC-like nuclease domains are inactivated, dead Cas9 (dCas9) is formed, which only retains its DNA-binding ability. These engineered Cas9 mutants, fused to other functional effectors or ligands, can be extensively used for specific gene targeting, activation, silencing, epigenetic regulation, and base editing.^38–40^ Besides SpCas9, other Cas9 proteins have also been designed, such as Cas9 derived from *Staphylococcus aureus* (SaCas9)^41^ and *Neisseria meningitidis* called Nme2Cas9.^42^ These Cas9 proteins, discussed later in this review, have comparable editing potential such as SpCas9 but are better suited for *in vivo* delivery, owing to their smaller size.

## CRISPR/Cas-Mediated *In Vivo* Gene Editing

A highly precise, robust, easily deliverable gene editing approach is required for safe *ex vivo* and *in vivo* clinical applications. During *ex vivo* editing, cells are first isolated, transfected with the appropriate gene editing toolbox, and then retransplanted into the patient.^43^ On a clinical scale, this is a time-consuming, strenuous, and expensive process, thereby questioning its broad accessibility to patients particularly in underdeveloped nations. Furthermore, *ex vivo* editing is largely limited to cells that can be isolated from a patient's body, modified *in vitro*, and then reinfused back into the patient, such as hematopoietic stem cells (HSCs) and immune cells, for example, T cells and natural killer (NK) cells. CRISPR/Cas-mediated *ex vivo* therapeutic gene editing has been extensively used for genetic diseases, such as sickle cell anemia, β-thalassemia, and chimeric antigen receptor (CAR)-T therapy.^44,45^ However, target cells implicated in the majority of the genetic diseases require *in situ* gene correction. Hence, *in vivo* gene editing is an ideal platform for treating various human genetic disorders. In the following section, we briefly highlight the rationale behind *in vivo* gene editing and CRISPR/Cas editing approaches devised for potential clinical use in monogenic diseases.

### Ideal candidates for *in vivo* gene editing

First, *in vivo* gene editing involves local or systemic delivery of the gene editing components into a patient, avoiding the tedious process of cell isolation, expansion, editing, and reinfusion.^46^ For example, the existing site-specific gene editing approaches to treat sickle cell disease include isolation of a patient's hematopoietic stem and progenitor cells, followed by either repairing the mutated hemoglobin gene (HBB)^47^ or inducing fetal hemoglobin expression,^48,49^ and finally, reinfusion of the corrected cells into the patient bone marrow.^50^ Advancements in *in vivo* technologies might alleviate the need for bone marrow transplantation, making the process less painful and economical.

Second, *in situ* gene modification is preferred for certain cell types that might lose their properties and function when artificially cultured, such as neurons. *Ex vivo* editing techniques also affect the viability of cells and result in poor engraftment, which is evaded during *in vivo* editing.

Third, in some monogenic disorders where a single gene impairment causes defects in the entire cell lineage, such as severe combined immunodeficiency,^51^ correcting HSCs generates healthy cells capable of differentiation, with a selective advantage over defective cells. As a result, a lower number of corrected cells are enough to attain therapeutic outcome. Since, the efficiency of *in vivo* editing is low, a selective advantage of the modified cells enhances the feasibility of this approach. Ideal candidates for *in vivo* gene editing are genetic disorders where allelic ablation of aberrant splice sites would help restore gene function, such as in β-thalassemia.^52^

Moreover, some genetic disorders that affect small organs, such as the ear and retina, require localized injection of the genome editing toolbox to the target organ, with limited distribution to other tissues. A localized delivery achieved by the route of administration or using tissue-specific promoters improves the feasibility of organ/tissue-specific genome editing *in vivo*.^53^ However, larger organs entail systemic injection for efficient targeting.

Finally, a conventional gene therapy approach involves replacement of the defective gene at the target locus. However, the low number of edited cells may not express adequate levels of the transgene necessary to alleviate the disease. This drawback can be resolved by delivery of the editing machinery to a native locus, “safe harbor” with high transcriptional activity, such as the serum albumin locus.^54^ This strategy established a versatile platform for therapeutic levels of protein expression, substituting the donor for each transgene.

### *In vivo* CRISPR/Cas applications

Most of the CRISPR/Cas-mediated therapeutic applications for monogenic disorders that are in clinical trials currently are *ex vivo* strategies. In recent years, *in vivo* gene editing studies that rely on both NHEJ and HDR pathways have emerged. Some of the recent applications of *in vivo* therapeutic genome engineering in preclinical and clinical studies are listed in Table 1. Here, we highlight some HDR-based precise gene modification studies *in vivo* using CRISPR/Cas that can be potentially translatable to human use in the future.

**Table 1.** *List of some of the recent therapeutic gene editing studies in* in vivo *preclinical and clinical models*

Disease	Target organ	Gene editing tool	Delivery system	Therapeutic modality	Reference
Hemophilia A and B	Mouse liver	ZFN	Systemic injection of AAV8	HDR- and HITI-dependent gene insertion	^135^
Hemophilia A and B	Mouse liver	ZFN	Systemic injection of AAV8	HR-based targeting of the genomic safe harbor, albumin	^136^
Hemophilia A and B	Mouse liver	ZFN	Systemic injection of AAV8	HDR-based corrective gene editing	^134^
Hunter's syndrome	Mouse liver	ZFN	Systemic injection of AAV2/AAV8	NHEJ- or HDR-mediated integration into albumin locus	^228^
HBV	Mouse liver	TALEN	Hydrodynamic injection of two plasmids encoding the editing machinery	Gene disruption of HBV sequences	^229^
HTI	Mouse liver	CRISPR/Cas9	Hydrodynamic injection of ssDNA donor template	HR-mediated point mutation correction	^56^
HTI	Mouse liver	CRISPR/Cas9	Intravenous injection of AAV2/8 and LNP	HDR-based point mutation correction	^58^
HTI	Mouse hepatocytes	CRISPR/Cas9	Hydrodynamic tail-vein injection of Cas9 and sgRNAs	NHEJ-based gene disruption	^230^
HTI	Mouse liver	CRISPR/Cas9 nickase	Hydrodynamic tail-vein injection of ABE system	ABE-mediated point mutation correction without HDR template	^61^
Transthyretin amyloidosis	Mouse liver	CRISPR/Cas9	LNP-mediated delivery of Cas9 and sgRNAs	NHEJ-based gene knockdown	^62^
AATD	Mouse liver	CRISPR/Cas9	Dual AAV delivery by intravenous (adult mice) and intraperitoneal injection (young mice)	HDR-based point mutation correction	^55^
AATD	Humanized mouse liver	CRISPR/Cas9	Intravenous injection of replication-deficient type5 Adenovirus	NHEJ-mediated mutant *AAT* disruption	^54^
Hemophilia B	Mouse hepatocytes	CRISPR/Cas9	Tail vein injection of AAV9 encoding liver-specific promoter	NHEJ-mediated gene inactivation of *F9* gene	^231^
DMD	Mouse muscle	CRISPR/Cas9	Intraperitoneal injection of AAV9	NHEJ-based mutant exon 23 excision	^70^
DMD	Mouse cardiac and skeletal muscle	CRISPR/Cas9	Intraperitoneal, intramuscular, and retro-orbital injection of AAV9	NHEJ-based mutant exon 23 skipping	^68^
DMD	Mouse cardiac muscle and skeletal myofibers	CRISPR/Cas9	Intramuscular and intravenous injection of AAV8	NHEJ-based mutant exon 23 excision	^69^
DMD	Mouse cardiac and skeletal muscle	CRISPR/Cas9	Intramuscular and intraperitoneal injection of AAV9	*DMD* gene restoration in ΔEx50 DMD mice by exon 51 skipping	^63^
DMD	Cranial tibialis muscles in dogs	CRISPR/Cas9	Intramuscular injection of AAV9	*DMD* gene restoration in ΔEx50 DMD canine model by exon 51 skipping	^64^
DMD	Anterior tibialis muscles in mouse	CRIPSR/Cas9 nickase	Intramuscular injection of AAV9	ABE-mediated point mutation correction	^71^
DMD	Mouse muscle	CRISPR/Cas9	Intramuscular and retro-orbital injection of dual AAV6	HDR-mediated dystrophin gene correction	^72^
Congenital muscular dystrophy type1A	Mouse muscle	CRISPR/dCas9	Intramuscular or systemic injection of AAV9	CRISPR activator-based gene upregulation	^232^
LCA type 2	Young adult eye	Human retinal pigmented epithelium-specific 65(RPE65) complementary cDNA under RPE65 promoter	Subretinal injection of rAAV2/2	AAV-mediated transduction	^233^
LCA10	Mouse retina	Self-inactivating CRISPR/Cas9	Subretinal injection of dual AAV5 vector	NHEJ-mediated intron deletion	^143^
LCA10	Humanized mouse eye	CRISPR/Cas9	Subretinal injection of AAV5	NHEJ-mediated aberrant splicing	^77^
Age-related macular degeneration	Adult mouse eye	CRISPR/Cas9	Subretinal injection of specific Cas9 RNP	NHEJ-based gene inactivation	^82^
Age-related macular degeneration	Adult mouse retina	CRISPR/Cas9	Intravitreal injection of single AAV9 vector	NHEJ-based gene inactivation	^80^
Retinitis pigmentosa	Transgenic mouse model with human *Rhodopsin* gene	CRISPR/Cas9	Electroporation of Cas9 and dual gRNAs in mouse retina	NHEJ-based gene knockdown	^234^
Retinitis pigmentosa	Rat retina	CRISPR/Cas9	Subretinal injection of gRNA followed by plasmid electroporation	NHEJ-based gene knockdown	^78^
Retinitis pigmentosa	Mouse retina	CRISPR/Cas9	Intravitreal injection of AAV9	NHEJ-based allele-specific targeting	^79^
Retinitis pigmentosa	Mouse retina	CRISPR/Cas9	Subretinal injection of gRNA and SpCas9	NHEJ-based allele-specific knockout	^83^
Retinitis pigmentosa	Mouse retina	CRISPR/Cas9	Dual AAV8 vector system by subretinal injection	NHEJ-based neural retina leucine zipper (*Nre*) knockdown	^85^
Retinitis pigmentosa	Mouse brain	CRISPR/Cas9	*In utero* electroporation in mouse embryos Subretinal injection in rats and intravenous injection of AAV8/9 in mouse	HITI-dependent gene correction	^87^
Retinitis pigmentosa	Mouse retina	CRISPR/Cas9	Subretinal injection of AAV8	NHEJ-mediated targeted *Nre* inactivation	^235^
Oxygen-induced retinopathy	Mouse eye	CRISPR/Cas9	Intravitreal injection of rAAV1	NHEJ-based mutant gene disruption	^81^
Primary open-angle glaucoma	Mouse eye	CRISPR/Cas9	Intravitreal injection of Adenovirus (Ad5)	NHEJ-based mutant gene disruption	^84^
Huntington disease	Mouse brain	CRISPR/Cas9	Stereotactic injection of AAV1	SNP-based allele-specific editing of *Htt* gene	^236^
Rett syndrome	Mouse brain	CRISPR/Cas9	Stereotactic injection of AAV1/2	NHEJ-based disruption of multiple genes	^103^
ALS	Mouse central nervous system	CRISPR/Cas9	Intramuscular injection of self-complementary AAV9	Insulin-like grown factor gene knockdown	^237^
ALS	Mouse spinal cord	CRISPR/Cas9	Systemic injection of AAV9	NHEJ-based gene disruption	^238^
Cardiac syndrome	Mouse heart	CRISPR/Cas9	Systemic injection of AAV9	NHEJ-based mutant gene knockdown	^146^
Dystrophic cardiomyopathy	Mouse heart	CRISPR/Cas9	Retro-orbital and intraperitoneal injection of AAV rh74	NHEJ-based mutant *Dmd* exon 23 excision	^73^
Lethal respiratory failure	Mouse fetus lung	CRISPR/Cas9	Intra-amniotic Ad vector delivery	NHEJ-based mutant gene disruption	^239^
Cancer	Programmed death1 ligand (PD-L1) tumor xenograft	CRISPR/Cas9	Lentiviral delivery	PD-1-deficient CAR-T cells	^240^
Genetic deafness	Mouse ear	CRISPR/Cas9	Inner ear injection of AAV2 Anc80L65 capsids	NHEJ-dependent mutant allele inactivation	^241^
HIV	HIV-infected humanized mouse spleen, brain, heart, lungs, and so on	CRISPR/Cas9	Intravenous injection of AAV-DJ/8	HIV-1 proviral DNA excision	^242^
HIV	Transgenic infected mouse spleen, liver, kidney, heart	CRISPR/Cas9	Tail-vein injection of rAAV9	HIV-1 proviral DNA excision	^243^
β-Thalassemia	Mouse model of human β-thalassemia	Triplex forming PNA	Intravenous injection of nanoparticles containing donor DNA	PNA-mediated gene editing	^91^
β-Thalassemia	Humanized mouse and thalassemia mouse blood cells	Transposase	Intravenous injection of HDAd5/35^++^ vector	Transposase-based gene integration	^92,93^
β-Thalassemia	Rhesus macaques	Transposase	Intravenous injection of HDAd5/35^++^ vector	Transposase-based gene integration	^95^
SCD	Humanized mouse and SCD disease model	CRISPR/Cas9 and transposase system	Intravenous injection of a bimodular HDAd5/35^++^ vector	Combined transposase-based integration and CRISPR/Cas9-mediated gene disruption	^96^

AATD, alpha-1 antitrypsin deficiency; AAV, Adeno-associated vector; ABE, adenine base editing; Ad, adenovirus; ALS, amyotrophic lateral sclerosis; CAR, chimeric antigen receptor; CRISPR/Cas, clustered regularly interspaced short palindrome repeats-associated Cas nuclease; dCas9, dead Cas9; DMD, Duchenne muscular dystrophy; gRNA, guide RNA; HBV, hepatitis B virus; HDR, homology-directed repair; HIV, human immunodeficiency virus; HITI, homology-independent targeted integration; HR, homologous recombination; HTI, hereditary tyrosinemia; LCA, Leber's congenital amaurosis; LNP, lipid nanoparticles; NHEJ, nonhomologous end-joining; PNA, peptide nucleic acids; RNP, ribonucleoprotein; SCD, sickle cell disease; sgRNA, single-guide RNA; TALEN, transcription activator-like effector nuclease; ZFN, zinc-finger nuclease.

#### Genetic liver diseases

Alpha-1 antitrypsin deficiency (AATD) patients suffer from progressive lung disease due to loss-of-function of AAT antiprotease activity and some patients suffer from liver toxicity due to gain-of-function of the mutant allele. CRISPR/Cas9-mediated editing and NHEJ successfully impaired mutant AAT and effectively ameliorated liver fibrosis in a humanized mouse model, thus supporting a potential therapeutic possibility of treating AATD patients.^55^ An additional study utilized coinjection of a dual adeno-associated vector (AAV): one encoding Cas9 and another expressing an AAT gRNA and an HDR donor template into the liver of a transgenic mouse model. This approach enabled precise AAT gene correction *in vivo* and partially restored wild-type AAT levels,^54^ making it a probable therapeutic option upon further optimization for use in humans. Hereditary tyrosinemia type I (HTI) is another genetic liver disease, caused by loss-of-function of fumaryl acetoacetate hydrolase (FAH), a key enzyme of the tyrosine catabolic pathway. CRISPR/Cas9-mediated HDR has successfully corrected FAH mutation by two methods: (1) A hydrodynamic injection of the gene editing components, which yielded a low correction rate^56^ and was tested in a clinical trial,^57^ and (2) systemic delivery of Cas9 mRNA by lipid nanoparticles (LNPs) and a single-guide RNA (sgRNA)/HDR template by AAV, which resulted in an initial *FAH* correction in more than 6% of hepatocytes.^58^ Moreover, a new-generation gene editing tool, base editing, which involves conjugating dCas9 with enzymes that catalyze direct conversion of A to G or C to T, ensues DNA base editing without causing any DNA breaks.^40,59,60^ Using an adenosine base editing (ABE) strategy via an LNP delivery containing sgRNA and a codon-optimized base editor was shown to restore *FAH* point mutation *in vivo*, eliminating the need for any DNA donor template.^61^ In addition, in a model of transthyretin amyloidosis, a single administration of LNP-mediated delivery of CRISPR/Cas9 along with chemically modified sgRNA facilitated efficient editing of the mouse transthyretin (*Ttr*) gene in the liver, and >90% reduction of TTR serum protein levels that persisted for at least 12 months.^62^ This study achieving clinically relevant levels of editing *in vivo* may be extended to provide human data in future.

#### Duchenne muscular dystrophy

*In vivo* editing studies have been explored in genetic muscular diseases, for example, Duchenne muscular dystrophy (DMD), characterized by progressive muscle weakness and premature death due to mutation in the dystrophin gene. A DMD mouse model exhibiting a similar deletion in the *Dmd* gene (ΔEx50) occurring in DMD patients was generated using CRISPR/Cas9. CRISPR/Cas9-induced single cut in the *dystrophin* gene of these mice and a gRNA that enables exon 51 skipping restores up to 90% dystrophin gene expression in skeletal and cardiac muscles.^63^ An important step toward clinical translation of therapeutic gene editing for DMD is using CRISPR/Cas9-mediated NHEJ to treat dogs with the ΔEx50 mutation, corresponding to a mutational “hotspot” in the human *DMD* gene. Systemic delivery of the gene editing apparatus in skeletal muscle provided 3–90% recovery, depending on the muscle type, and treated dogs revealed improved muscle histology.^64^ Although this proof of principle study in the canine disease model has the potential to bridge the gap between mice and humans, there are some issues in large animal editing which needs further attention. Limited sample size, age of injection, treatment duration, characterization of the treatment results, safety and ethical concerns as discussed in earlier reports^65–67^ needs to be addressed in future.

Besides exon skipping, other groups have utilized AAV-based local and systemic delivery of CRISPR/Cas9 editing components to adult and neonatal DMD mouse models for removing the mutation in exon 23, resulting in partial recovery of functional dystrophin in skeletal myofibers and cardiac muscle.^68–70^ Moreover, local delivery of ABEs consisting of engineered adenine deaminase, and an SpCas9 nickase helped correct a nonsense mutation in a DMD mouse model.^71^ An ideal therapy for a chronic disease such as DMD should ensure a lifelong, sustained restoration of dystrophin in the heart and skeletal muscle. To this end, a single-dose AAAV-CRISPR therapy that leads persistent alleviation of the disease phenotype is required. Successful results from short-term studies^68,70,72,73^ prompted researchers to test the long-term restoration of the *DMD* gene. Systemic delivery of an AAV9 vector encoding SaCas9 and gRNA targeting introns 22 and 23 restored dystrophin expression, thereby improving skeletal and cardiac muscle function for 18 months in dystrophic mice.^74^ Another approach for attaining enduring gene therapy for DMD would be editing muscle stem cells (MuSCs) using CRISPR. Since the self-renewing MuSCs, also known as satellite cells, regenerate skeletal muscle in response to tissue damage, correcting these cells would enable long-term therapeutic gene editing. CRISPR-edited MuSCs from dystrophic mice, when engrafted in a dystrophin null mouse, showed increased dystrophin expression and successful renewal.^75,76^ These studies taken together demonstrate that with further development, *in vivo* gene editing approaches will be clinically useful for treating DMD.

#### Retinal disorders

A hallmark study that recently entered clinical trial uses *in vivo* CRISPR/Cas9 delivery for treating congenital blindness in patients. Leber congenital amaurosis (LCA) is a rare, debilitating monogenic disease resulting in vision loss in childhood, with no available treatment. A biallelic loss-of-function mutation in the *CEP20* gene is responsible for this severe retinal dystrophy. Editas Medicine has developed a therapy named, EDIT-101, which delivers SaCas9 directly to remove the intronic IVS26 mutation in the *CEP20* gene, implicated in aberrant splicing, thereby restoring functional CEP20 levels in human cells and humanized CEP20 mice.^77^ A clinical trial of EDIT-101 by Allergan and Editas Medicine paves the way for a prospective curative strategy for treating congenital blindness using an *in vivo* approach.

Ideally, HDR-based precise gene correction can repair the genetic mutations implicated in inherited retinal disorders. Since HDR mainly occurs in mitotic cells, the postmitotic nature of most retinal cells limits the HDR efficiency. Hence, a majority of the *in vivo* gene therapy approaches for retinal dystrophies rely on the CRISPR-Cas-mediated NHEJ pathway.^78–86^ Another genome editing strategy, namely, homology-independent targeted integration (HITI), was utilized to successfully knock in exon 2 of *Mertk* (MER/AXL/TYRO3 receptor kinase) gene, thereby protecting from retinal degeneration.^87^ HITI exploits the NHEJ repair mechanism and enables targeted transgene insertion without the need of an HR donor template in dividing and nondividing cells.

#### *In vivo* editing of stem cells and immune cells

Most of the HSC gene therapies involve removal of the patient's stem cells, their expansion followed by gene correction using editing machinery and then reintroduction to the patient's body. Although this approach has been used in ongoing clinical trials, they are associated with limitations, discussed earlier in this review. Another disadvantage of the *ex vivo* approach is that reinfusion of the edited cells into the bone marrow requires the patient to undergo chemotherapy. Recently, one of the ongoing clinical trials for sickle cell disease, initiated by Bluebird Bio, has come to a halt after two patients, who received the *ex vivo* gene therapy for SCD, were diagnosed with acute myeloid leukemia (AML) and myelodysplastic syndrome (MDS).^88^ Previously, in 2018, another patient in the same trial was diagnosed with MDS, likely due to the adverse effects of chemotherapy pretreatment. Whether these two new cases can be attributed to chemotherapy or insertional oncogenesis triggered by the lentiviral vector used in the trials is still elusive and needs further examination. *In vivo* editing bypasses the time-consuming, expensive, and laborious process of *in vitro* handling of HSCs as well as the DNA damaging chemotherapy during reinfusion. *In vivo* approaches either include a direct modification of HSCs in the bone marrow by an intraosseal injection, or a systemic injection of delivery vehicles that act on HSCs mobilized into peripheral blood, followed by their re-engraftment into bone marrow. Previous studies have reported successful lentiviral-mediated gene transfer in T cells^89^ and in HSCs by direct intraosseal injection in mice, demonstrating high levels of transduction in bone marrow cells.^90^ An *in vivo* HSC gene editing study was reported in thalassemic mice injected with nanoparticles containing triplex-forming peptide nucleic acids and a single-stranded homologous DNA donor, in combination with the stem cell factor. This editing strategy showed almost 7% editing frequency in the bone marrow, sufficient to ameliorate the disease phenotype.^91^ An alternative *in vivo* gene therapy strategy involved mobilization of hematopoietic stem and progenitor cells (HSPCs) from the bone marrow into peripheral blood, followed by an intravenous injection of integrating, helper-dependent adenovirus (HDAd5/35^++^) vector system that targets human CD46 expressed on nascent HSCs. This transposase-based integration system achieved stable fetal γ-globin expression in CD46-transgenic and thalassemia mouse models.^92–94^ This method when tested in rhesus macaques demonstrated stable HSC transduction, thereby improving its feasibility in human HSC gene therapy.^95^ Besides thalassemia, this approach has been recently used to correct the sickle cell phenotype. An HDAd5/35^++^ vector encoding two cassettes, one containing the CRISPR/Cas9 machinery and the other encoding the therapeutic fetal γ-globin transgene, was administered by an intravenous injection in an SCD mouse model. A combination of these two cassettes induced expression of the fetal γ-globin gene and ameliorated the disease phenotype.^96^ Despite the promising results, the high titer of the immunogenic adenoviral vectors might hinder clinical trials. An AAV vector delivery system may be a safer and more efficient alternative for *in vivo* HSC gene editing. Recombinant tyrosine mutant AAV6 vectors displayed high transduction efficiency and robust transgene expression in human HSCs *in vitro* and in a mouse xenograft model *in vivo*.^97–99^ In addition, AAV8-mediated transduction of immune cells, such as T cells, B cells, macrophages, and dendritic cells, was achieved *in vivo* after systemic injection in mice,^100^ thereby spurring the development of these vectors for *in vivo* immunotherapies. Another recent gene editing strategy using base editors delivered by HDAd5/35^++^ vectors revealed efficient HSPC transduction and stable γ-globin expression in transgenic mice, strengthening its immense potential for *in vivo* gene therapy for hemoglobinopathies.^101,102^ Future studies exploring HDR-based *in vivo* HSC editing will enrich the field of hematopoietic gene therapy.

#### Brain disorders

NHEJ-based editing triggered by CRISPR/Cas9 system has been extensively studied in brain regions *in vivo*.^103–107^ Compared with NHEJ, the low efficiency of the HDR pathway in the postmitotic neurons makes precise gene correction difficult in these cells. To overcome this, HITI has been used to achieve targeted insertion of the desired donor sequence *in situ*.^87,108^ This can be used to create knockin reporter systems for cell tracking in live animals, useful for studying neuronal circuits and brain functions. Moreover, some studies suggest that neuronal progenitors retain their ability to trigger HDR *in vivo*.^109^ A rapid *in utero* electroporation method to deliver the editing components into neuronal progenitors *in vivo* enabled successful HDR editing in the mouse embryonic brain.^110–112^ HDR-facilitated gene editing has been shown in postmitotic neurons as well. A combination of CRISPR/Cas9 and AAV-mediated donor DNA delivery enabled HDR editing *in vivo* along with the insertion of a reporter tag in the brain regions. This strategy, known as vSLENDR (viral-single-cell labeling of endogenous proteins by CRISPR/Cas9-mediated HDR), was adapted to conduct precise gene modification by HDR in any regions of the brain.^113^

Some of the existing *in vivo* therapeutic gene editing studies are summarized in Table 1. Despite these promising studies listed above, there still exists a lacuna between animal studies and applications in humans, further emphasizing the need for improved *in vivo* editing, discussed in the next section.

### *In vivo* gene editing clinical trials

The gene editing landscape is evolving rapidly with the advancement of several therapeutic gene editing studies to clinical trials. Most of the ongoing trials are focused on gene modification *ex vivo* and have been reviewed.^114,115^ Currently, the *ex vivo* gene editing preclinical and clinical trials primarily involve alteration of T cells to disrupt gene expression for treating HIV,^116–120^ engineering T cells for cancer immunotherapy,^121–124^ and modification of HSCs for treating hemoglobinopathies, such as β-thalassemia and sickle cell anemia.^18,48,125–127^ Clinical trials for β-thalassemia and sickle cell anemia using ZFN- and Cas9-mediated disruption of the fetal globin repressor BCL11A in HSCs *ex vivo* are ongoing (NCT03432364, NCT03653247, NCT03655678, and NCT03745287).

*In vivo* therapeutic gene editing approaches have also advanced into clinical trials, summarized in Table 2. ZFN-, TALEN-, and Cas9-based trials for treating cervical cancer have been registered. These approaches target the E6 and E7 genes of human papilloma virus (HPV), the causative agent of cervical cancer.^128^ Although HPV vaccines are available now, they do not confer treatment for cervical cancer patients. Nonviral delivery of ZFNs, TALENs, or CRISPR/Cas9 achieved targeted disruption of the E7 oncogene, resulting in reduced tumor growth in mouse models.^129–131^ Besides nonviral delivery methods, AAV-dependent delivery of Cas9 targeting E6 and E7 viral genes showed encouraging results in xenograft models,^132,133^ reflecting its therapeutic potential for cervical cancer. In addition to the *in vivo* trials on cervical cancer, ZFN-mediated gene editing has been used to treat hemophilia.^134–137^ ZFN-based gene correction of factor IX, α-l-iduronidase, and iduronate-2 sulfatase have proceeded to clinical trials for hemophilia, mucopolysaccharidosis type I (MPS I), and MPS II, respectively. Initial results from the MPS II trial affirm the safety of this approach. More robust, second-generation ZFNs will be used in future to increase the editing efficiency in human liver cells.^138^ The most recent CRISPR/Cas9-mediated trial of EDIT-101 (NCT03872479) led by Allergan and Editas Medicine utilizes AAV-based delivery to the eye, discussed in the Retinal Disorders section. Subretinal injection of the CRISPR/Cas9 editing machinery in mouse and primates has shown gene editing at therapeutic levels, restoring normal expression of *CEP290* gene in patients suffering from congenital blindness.^77^ So far, the current registered *in vivo* clinical trials target tissues that are readily accessible, such as the cervix, liver, and eye. The continuous advancement of *in vivo* gene editing technologies will undoubtedly spur the development of more clinical studies to treat a myriad of human genetic diseases in future.

**Table 2.** *List of the* in vivo *gene editing clinical trials*

Disease	Gene editing tool	Therapeutic strategy	Phase	Organization	NCT number
Cancer caused by HPV	ZFN	Polymer gel-based plasmid delivery enabling ZFN-based deletion of E7 oncogene in HPV16 and HPV18	I	Huazhong University of Science and Technology, China	NCT02800369
HPV-related cancer	TALEN	Polymer gel-based plasmid delivery enabling TALEN-based deletion of E6 and E7 oncogene in HPV16 and HPV18	I	Huazhong University of Science and Technology	NCT03226470
HPV-related cancer	TALEN	Polymer gel-based plasmid delivery enabling TALEN-based deletion of E6 and E7 oncogene in HPV16 and HPV18	I	First Affiliated hospital, Sun Yat-sen University, China	NCT03057912
HPV-related cancer	CRISPR/Cas9	Polymer gel-based plasmid delivery enabling TALEN-based deletion of E6 and E7 oncogene in HPV16 and HPV18	I	First Affiliated hospital, Sun Yat-sen University	NCT03057912
Hemophilia B	ZFN	Factor IX gene insertion into albumin locus of hepatocytes	I	Sangamo Biosciences	NCT02695160
MPS type I	ZFN	*IDUA* gene insertion into albumin locus of hepatocytes	I	Sangamo Biosciences	NCT02702115
MPS type II	ZFN	*IDS* gene integration into albumin locus of hepatocytes	I	Sangamo Biosciences	NCT03041324
LCA10	CRISPR/Cas9	SaCas9-mediated removal of intronic IVS26 mutation in *CEP20* gene	I	Allergan and Editas Medicine, Inc.	NCT03872479

U.S clinical trial data from https://www.clinicaltrials.gov/

HPV, human papilloma virus; *IDS*, iduronate-2 sulfatase; *IDUA*, α-l-iduronidase; MPS, mucopolysaccharidosis; SaCas9, *Staphylococcus aureus* Cas9.

## Limitations Associated With CRISPR/Cas-Based *In Vivo* Therapeutic Gene Editing

**Figure 2.**
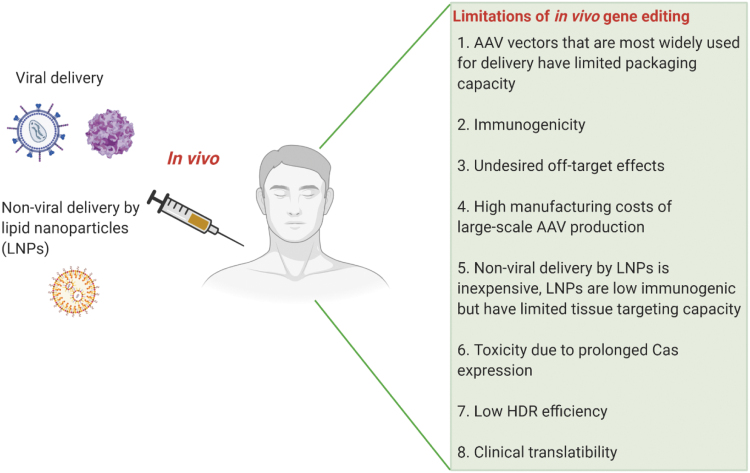
Schematic showing limitations of *in vivo* gene editing. *In vivo* gene therapy involves direct injection of the editing machinery into the patient by viral or nonviral delivery methods. Limitations associated with this approach are listed here. Created with BioRender.com

In this section, we briefly describe some of the unmet challenges and possible strategies to alleviate them, facilitating the clinical utility of therapeutic *in vivo* gene editing. A summary of the limitations associated with *in vivo* gene therapy is depicted in Fig. 2.

### *In vivo* delivery

The key step that determines the clinical utility of genome editing is the efficient and safe delivery of the editing toolbox, including CRISPR/Cas enzymes, sgRNA, and repair template, to the target cells. Cas enzyme can be delivered to the cells in several formats: plasmid DNA encoding *Cas* gene, Cas mRNA or protein. These are coupled with the appropriate sgRNA. Electroporation of cells with a preformed Cas protein and sgRNA RNP complex is the preferred form of delivery in *ex vivo* gene editing.^139^ Although electroporation has been used to deliver Cas9 to animal zygotes^140,141^ and skeletal muscle in mice,^142^ the high-voltage shock required to permeabilize cells is toxic and may not be favorable for a broad range of applications*. In vivo* delivery is more challenging and requires carriers that have high specificity, low cytotoxicity, and rapid clearance of Cas enzyme after gene editing. Overcoming these challenges to enhance the clinical prospects of *in vivo* gene editing has stimulated the development of viral and nonviral delivery systems. Among the viral delivery methods, which include lentiviruses, adenoviruses, and AAVs, the most widely used for *in vivo* delivery of CRISPR/Cas systems is AAVs.

AAVs are relatively nonimmunogenic, demonstrate capsid variant-dependent tissue specificity than other viral vectors, and long-term transgene expression without the necessity of genomic integration. AAV-mediated delivery of CRISPR/Cas9 has been used successfully in gene therapy for monogenic diseases, such as DMD,^68–70,72^ retinal impairments,^80,85,143,144^ and liver,^41,58,145^ heart,^146–148^ and lung disorders.^149^ The single-stranded AAV genome and its unique inverted terminal repeats play an essential role in precise gene targeting. The ssDNA of rAAVs accommodates long homology arms, encodes selection markers, and has low NHEJ-based integration rates, thereby making it a *bona fide* HDR template. While the CRISPR/Cas components can be delivered by different methods that do not necessarily require AAVs, the HDR donor is often delivered by single-stranded AAVs.^150,151^ Although AAVs serve as a favorable delivery vehicle for CRISPR/Cas, their packaging capacity is limited only to 4.7 kb. Using a dual-vector system, one expressing a gRNA and an HDR repair template and the second AAV encoding SpCas9 gene (4.2 kb), for HDR-mediated gene correction can avoid the packaging size limitation.^145,152^ Alternate approaches for precise gene correction *in vivo*, such as base and prime editing, also need dual vectors to accommodate effectors fused to Cas9.^153–157^ However, the target cells need to uptake both the vectors together, thereby affecting editing efficiency. Also, a high AAV dose required in the dual-vector studies might raise safety concerns during clinical translation, considering the recent consequences of a high-dose AAV therapy in human trials.^158,159^ Smaller Cas9 orthologs, such as SaCas9, NmeCas9, or Cas9, from *Campylobacter jejuni* can be combined with the gRNA and donor template in a single AAV vector to eliminate the packaging issue.^42,80,160,161^ A novel all-in-one recombinant AAV vector encoding Nme2 Cas9 along with two sgRNAs was engineered to alleviate the disease phenotype in an HTI mouse model. To fulfill the need of a single-AAV for precise gene modification by HDR, this system was further updated. A self-inactivating single AAV vector, encoding Nme2 Cas9, a single sgRNA, and an HDR donor flanked by Nme2 Cas9 target sites, was designed. Self-cleavage during packaging was circumvented by including an anti-CRISPR protein (ACR).^162^ Precise HDR-based therapeutic editing at clinically relevant levels were obtained in disease models of HTI and MPS I.^163^ Newly discovered CRISPR/Cas systems, such as the hypercompact CasΦ, which is half the size of SpCas9, show similar efficiency and selectivity and have the potential of circumventing the size limitation of AAV-based delivery.^164^ Second, the tissue tropism of AAVs needs further improvement to minimize any undesired side effects of CRISPR/Cas in other tissues. AAV capsids can be engineered to use tissue-specific promoters,^72,103,147^ or with improved capsid variants^165–167^ or to increase target tissue specificity or transduction efficacy *in vivo* by incorporating ligands that bind to receptors on target cells.^168^ Other constraints of AAVs such as delivery carriers include immunogenicity,^169^ high viral titers beyond clinically accepted levels for obtaining therapeutic editing, and expensive manufacture and scalability for clinical use.^170^

The administration route of AAV vectors also affects the efficiency and specificity of *in vivo* gene editing. Selection of an optimum injection route depends on the target tissue, tissue-specific promoter, and AAV capsid variant. For example, a systemic intravenous injection is the preferred delivery route for editing genes implicated in liver disorders since most of the AAVs accumulate in the liver.^145,171^ However, a localized injection, such as subretinal or intravitreal injections, is favored while administering AAVs containing CRISPR/Cas components into the mouse retina.^85,143,144^ Although most of the *in vivo* methods have the potential to be extended to human studies, the high dosage required to achieve clinically relevant editing levels questions their translatability to humans.

Despite the widespread use of AAV-mediated delivery systems, the limitations discussed above prompted the development of nonviral carriers for delivering CRISPR/Cas *in vivo*. Cationic LNPs have been used to deliver CRISPR/Cas9 RNP to mouse liver to obtain therapeutically relevant gene editing *in vivo*.^62^ This study resulted in 70% editing efficiency with a single dose and yielded effective results in rats, validating its preclinical potential.^62^ Other groups that have reported *in vivo* editing using nonviral delivery systems in the liver have efficiencies ranging from 3.5% of hepatocytes^172^ to 35% editing after four systemic doses.^173^ A recent study shows that LNPs were able to effectively deliver gRNA and Cas9 mRNA to splenic endothelial cells, thus identifying new accessible target cells *in vivo*.^174^ Despite this method being inexpensive, rapid, and easy,^175^ the LNPs show some evidence of toxicity.^58,176^ A delivery system, consisting of gold nanoparticles conjugated to DNA and assembled with polymers that disrupt endosomes, can deliver Cas9 RNP and donor DNA to correct *Dmd* gene mutation in mice, with negligible off-target effects.^177^ Further advancements in nanoparticles, nanowires, and cell-based delivery methods are crucial for therapeutic *in vivo* genome editing.^178,179^

### Off-target effects of CRISPR/Cas

Precise and accurate gene modification at the desired target site is imperative for therapeutic genome editing. Although the CRISPR/Cas system is known to be more precise in comparison with the other nucleases, it still exhibits off-target cleavage activity. Off-targeting occurs due to nonspecific CRISPR/Cas-induced DNA cleavage at sites other than the actual target and may result in deleterious effects, such as malignant transformation.^180^ Some of the *in vivo* gene therapy studies revealed minimal or no off-target editing at the predicted sites, which is reassuring.^58,69,70,145^ However, the possibility of off-target editing beyond the predicted sites requires the design of an unbiased genome-wide sequencing method. Several cell-based genome-wide sequencing tools, such as CHIP-seq^181^ and Digenome-seq,^182^ have aided in the identification of unpredictable off-target mutations *in vitro*. However, these *in silico* tools cannot be directly applied to identify undesirable genomic sites for *in vivo* editing. A two-step strategy, named “verification of *in vivo* targets” (VIVO), has been developed to first identify potential off-target locations using CIRCLE-Seq, and then confirm any alteration of these sites following CRISPR/Cas9 *in vivo* genome editing.^183^ This powerful *in silico* tool allows identification of off-target mutation sites *in vivo,* vital for designing the most specific gRNA that acts on the desired genomic sites.

Besides optimizing gRNA design, reducing long-term expression of Cas9 is another way of minimizing off-target effects. Delivery of short-lived Cas9 protein instead of the Cas9 gene,^184^ using a self-limiting CRISPR/Cas system for conditional genome editing,^143^ or inducible Cas9 variants,^185–187^ diminishes duration of Cas9 exposure, thereby impeding its off-target effects. In addition, a self-inactivating AAV-CRISPR system containing a gRNA that cleaves Cas9 coding sequence can eliminate the Cas9 protein *in vivo* without affecting targeted editing efficiency, thereby alleviating the problems associated with long-term Cas9 expression.^163^ Furthermore, LNP-mediated delivery of Cas9mRNA^58^ and extracellular vesicle (EV)-mediated delivery of CRISPR-Cas9 RNPs minimize off-target cleavage by limiting prolonged Cas9 exposure.^188–193^ A recently developed all-in-one EV-based delivery system known as, NanoMEDIC (nanomembrane-derived EVs for the delivery of macromolecular cargo), promotes on-target gene editing both *ex vivo* and *in vivo*.^194^ Alternate approaches to circumvent the off-target effects include editing methods that do not require double-stranded cleavage by CRISPR/Cas9. For example, dCas9 fused to transcriptional activators or repressors engaged in CRISPR activation and interference studies has higher specificity.^38,39^ Base editors ensuing RNA-programmed DNA base editing without causing any DNA break also restrict undesirable off-target editing.^60,195^ Another way to reduce off-target effects is using anti-CRISPR proteins that regulate dCas9 activity and generate cells resistant to nonspecific gene modifications.^196–198^ The robustness and specificity of these techniques *in vivo* still need to be studied comprehensively before their clinical use.

### CRISPR/Cas immunogenicity

There are two predominant issues regarding the immunogenicity of CRISPR gene editing, one is the toxicity of Cas9 expression and the other is the preexisting immunity against Cas9. Toxicity associated with prolonged Cas9 expression and ways to alleviate them^58,143,163,184–187^ have been discussed in the previous section. A humoral and cellular immune response was elicited against SaCas9 only in adult mice receiving AAV-CRISPR based gene therapy for DMD. However, neonates did not exhibit any immune response against the bacteria derived SaCas9 proteins.^199^ Humanized Cas9 protein might also be less immunogenic reducing its potential toxicity. Host immune responses against Cas9 may hinder *in vivo* therapeutic gene editing. Since the most widely used Cas9 orthologs, SpCas9 and SaCas9, are both derived from bacterial species that frequently infect humans, it is likely that humans will harbor preexisting immune responses against them. As expected, preexisting immunity of anti-Cas9 IgG antibodies was found against SaCas9 and SpCas9 in healthy human adults.^200^ Reactive T cells against SpCas9 were also detected in humans.^201^ Edited cells may be eliminated due to CRISPR/Cas-triggered immune response. In one study, preexisting immunity to Cas9 led to a high percentage of cytotoxic CD8^+^ T cells in mouse liver, resulting in removal of edited cells.^202^ Development of methods for diminishing the immunogenicity of CRISPR/Cas toolbox requires further attention.

### HDR efficiency

Precise gene correction for monogenic disorders is achieved by HDR. However, the efficiency of HDR-dependent precision gene modification is lower compared with other competing repair pathways, such as NHEJ. HDR occurs mostly in mitotic cells, making it difficult to improve its efficiency to match therapeutic levels. Although the editing efficiency for different diseases varies, a higher efficiency usually augments the therapeutic outcomes. Optimum and rational designing of HDR donors,^203^ increasing sequence similarity between the donor template and target cleavage sites,^204^ and inhibiting NHEJ pathways^205,206^ are some of the advancements that enhance HDR efficacy. HITI strategies can also be used to obtain targeted integration of the desired transgene to facilitate *in vivo* gene therapy.^87,108,207^ In addition, base editors^40,208^ and prime editors^209^ that allow precise gene editing, independent of DNA repair pathways, can potentially cure several genetic diseases.

## Nuclease-Independent Gene Targeting as an Alternate Editing Approach

**Figure 3.**
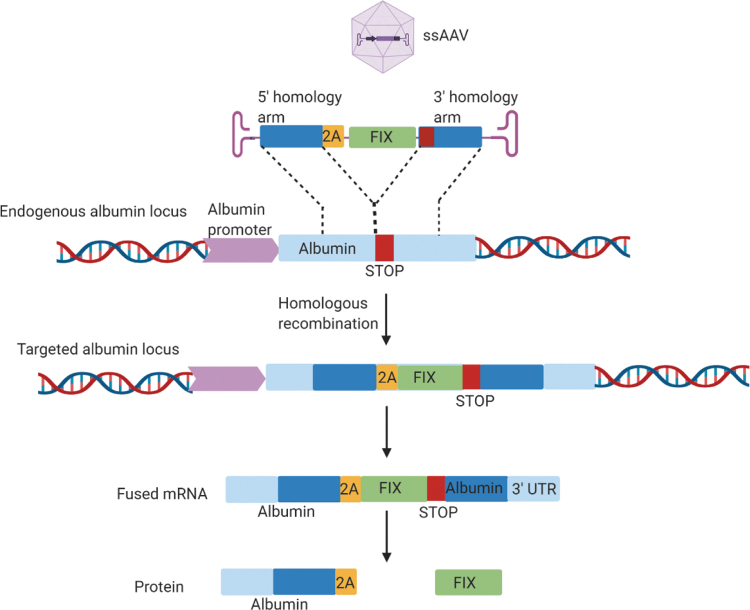
Schematic showing promoterless nuclease-free editing at the albumin locus. Recombinant AAV8 vector containing a promoterless codon-optimized human coagulation FIX sequence I (*green*), preceded by the 2A peptide (*yellow*) and flanked by albumin homology arms (*dark blue*) that covers the albumin stop codon (*red*), is designed. Homologous recombination results in integration of the rAAV8 vector into the endogenous albumin locus (*light blue*) and generates a chimeric bicistronic mRNA, which is translated into two distinct proteins, albumin and FIX, due to the ribosomal skipping. Adapted from Barzel *et al.*^210^ AAV, adeno-associated vector; FIX, factor IX. Created with BioRender.com

The risks associated with nuclease-dependent gene targeting, as discussed above, include the inadvertent prolonged expression of Cas9, resulting in potential off-target effects. To eliminate this problem, a nuclease-free gene targeting strategy based on HR was developed by Barzel *et al*.^210^ In this method, a recombinant AAV8, containing a promoterless, codon optimized FIX coding sequence, flanked by sequences homologous to the mouse albumin locus, was designed. A porcine teschovirus-1 2A-peptide (P2A) encoding sequence preceding the *F9* gene sequence was used for ribosomal skipping to ensure that the bicistronic Alb-FIX mRNA transcribed from the endogenous Alb promoter is translated into functional albumin and FIX proteins (Fig. 3). This alternative *in vivo* nuclease-free editing approach attained FIX expression at therapeutic levels to partially correct the spontaneous bleeding phenotype in hemophilic mice.^210^ This forms the basis of LogicBio's proprietary GeneRide technology and utilizes HR-guided precise and targeted *in vivo* gene editing, eliminating the need for vector-driven promoters and engineered nucleases.^211^ In addition, a versatile system for *in vivo* selection and expansion of gene-modified hepatocytes, irrespective of genetic background, has been established using GeneRide.^212^ Another recent study published by Homology Medicines revealed the proficiency and specificity of HR-mediated, nuclease-free gene insertion in mouse liver containing human cells using AAVs derived from human HSCs.^213^

Although this method is less efficient compared with nuclease-mediated editing, it can work well provided there is a selective advantage of the edited cells.^211^ These examples using the nuclease-independent *in vivo* gene targeting strategy herald an overall safe, robust, and precise avenue for gene therapy.

## Perspective

The advent of CRISPR/Cas technology has undoubtedly fostered the development of therapeutic gene editing for a multitude of genetic diseases. Currently, there are several ongoing clinical trials of nuclease-dependent gene therapy, with the hope to ameliorate monogenic disorders, such as hemoglobinopathies and retinal dystrophy, among others. Some examples include the *ex vivo* CRISPR-mediated gene therapy for β-thalassemia and sickle cell anemia, known as CTX001, currently in clinical phase 1/2 trials. SB-FIX by Sangamo Therapeutics is an *in vivo* gene therapy treatment that uses AAVs to deliver ZFNs to correct the factor IX gene for treatment of hemophilia B. Another milestone study is the first phase 1 clinical trial NCT02793856 on CRISPR/Cas9-based PD-1 gene knockout in T lymphocytes from metastatic nonsmall-cell lung cancer patients. Furthermore, Allergan and Editas Medicine are conducting a clinical trial of a candidate genome editing therapy, EDIT101, to cure LCA (Table 1). These studies reinforce the tremendous potential of engineered nucleases for treating genetic diseases.

In addition, gene editing has been applied to cancer immunotherapy, and one promising area that has garnered great interest is the development of allogeneic CAR-T therapy. ZFN- and TALEN-mediated gene editing has enabled the generation of allogeneic tumor-associated antigen-specific CAR-T cells, with negligible T cell immune response and graft-versus-host disease.^25,124,214,215^ Furthermore, CRISPR/Cas9 triggered faster and easier multiplex gene editing in CAR-T cells, which exhibited CD19-specific antitumor activity in a lymphoma xenograft mouse model.^216^ Allogeneic universal T cells were generated using a one-shot CRISPR technique with multiple sgRNAs in a CAR lentiviral vector that simultaneously depleted the endogenous T cell receptor and HLA 1, thereby eliminating rapid rejection from the host immune system.^217^ Recent studies use CRISPR/Cas9 to specifically inhibit immune receptors^218,219^ to enhance the generation of “universal” CAR-T cells, which might be an effective treatment for AML and other malignancies. The efficacy and safety of the CRISPR/Cas9-edited CAR-T cells in clinical studies need evaluation. The ongoing clinical trials of the modified universal CAR-T cells have been reviewed.^115,220,221^ CRISPR/Cas editing machinery eliminated some of the limitations associated with CAR-T immunotherapies, thereby enhancing efficiency of off-the shelf CAR-T cells and minimizing their toxicity.^222^ Overall, these findings reflect the immense prospective of gene editing as a robust platform to generate CAR-T cells as an off-the shelf therapy. Moreover, over 300 clinical trials are ongoing across the globe for improving CAR activity and broadening their clinical applications.^89^ Yescarta for adult diffused B-cell lymphomas and Kymriah for pediatric acute lymphoblastic leukemia, approved by the US Food and Drug Administration (FDA) and European Medicines Agency (EMA) have hit the market. Generating human CAR-T cells directly *in vivo* will be very useful in circumventing the expensive and laborious *ex vivo* production of CAR T cells, rendering them more accessible to patients worldwide. In a recent study, a CD8 targeted lentiviral-based single systemic injection of CD19-CAR-T cells into humanized immunodeficient mice generated *in vivo* CAR-T cells, that successfully eliminated human B cells. This study resulted in a cytokine storm in humanized mice and further preclinical testing is required to test the feasibility of the approach.^223^ Evaluation of the anti-tumoral activity of these *in vivo* generated human CAR-T cells was done in T cell engrafted immunodeficient mouse models for preclinical testing of CAR-T cells.^224^ Next, successful *in vivo* generation of CD19 CAR-T cells in CD4+ T cells was reported that had the ability to eliminate the CD19 + cells and tumor cells in mice, highlighting the relevance of *in vivo* CAR-T cell therapy.^225^ Although these results look promising, whether the *in vivo* generated CAR-T cells match the efficacy of the *ex vivo*-generated CAR-T cells needs further validation. Assessments in large animal models is required before the commencement of an *in vivo* CAR-T cell therapy a clinical trial in future.

## Conclusion

Besides gene editing, the CRISPR/Cas toolbox has also been used for gene regulation, epigenetic modification, drug development, and precision medicine providing personalized therapies based on specific targets and diagnostics, extensively reviewed elsewhere.^226,227^

In general, the CRISPR/Cas system provides a precise platform for *ex vivo* and *in vivo* therapeutic gene editing against debilitating genetic diseases. So far, *ex vivo* editing has been predominantly used to treat hemoglobinopathies, cancers, and immune cell disorders. Since a wide range of genetic diseases require *in situ* gene modification, *in vivo* gene editing has the tremendous potential to treat them. While the *in vivo* approach minimizes the risk of graft-versus-host disease and immunosuppression, there are existing barriers that hinder its clinical translatability. One of the primary bottlenecks of *in vivo* gene therapy is the targeted delivery of the editing machinery. Currently, AAV vectors are the most popular delivery tools for introducing the transgene and CRISPR/Cas system to target organs. However, the limited packaging capacity, off-target effects, and high production costs are some of the limitations of AAV vector delivery. Alternatively, nonviral delivery methods that allow flexible packaging ability, ease of manufacturing, and have low cytotoxicity have shown promise. Another concern that affects the efficacy and safety of the CRISPR/Cas-mediated *in vivo* editing is the off-target effects. Further progress in the delivery of viral and nonviral delivery vectors, and CRISPR/Cas components, is necessary to attain clinically relevant levels of gene editing *in vivo*.

Overall, the *in vivo* studies demonstrate the ability of both nuclease-mediated and nuclease-free editings as potent gene therapy tools. However, obstacles such as off-target effects, optimum delivery vehicles, HDR efficiency, and immunogenicity of the editing components have not been completely resolved. With innovative gene editing advancements in the future, these bottlenecks will be surmounted, thus bringing *in vivo* gene editing closer to human therapies.
